# Adverse Pregnancy Outcomes in Women With Immune Abnormalities: Machine Learning Model Development and Validation Using First-Trimester Sonographic Features

**DOI:** 10.2196/84087

**Published:** 2026-05-13

**Authors:** Shijin Xu, Yan Jiang, Qiaoyu Zhang, Qiao Xu, Qinxin Wang, Chang Zhou, Rong Liu, Yun Liu

**Affiliations:** 1 Department of Ultrasound Imaging, The First College of Clinical Medical Science, China Three Gorges University & Yichang Central People’s Hospital, No. 183 Yiling Road, Yichang, 443000, China, 86 19071786542; 2 Department of Ultrasound, Aerospace Center Hospital, Beijing, China

**Keywords:** machine learning, ML, ultrasound features, immune abnormalities, pregnancy outcomes

## Abstract

**Background:**

The maintenance and progression of pregnancy rely on immune homeostasis at the maternal-fetal interface. However, pregnancy complicated by autoimmune abnormalities can disrupt this balance and significantly increase the risk of adverse pregnancy outcomes (APOs).

**Objective:**

This study aimed to (1) develop an interpretable predictive tool for APOs in patients with immune abnormalities and (2) interpret the models using Shapley additive explanations (SHAP) values.

**Methods:**

This study retrospectively analyzed clinical data from 288 patients with autoimmune abnormalities at Yichang Central People’s Hospital between 2019 and 2024. Feature selection was performed using both the Boruta algorithm and Least Absolute Shrinkage and Selection Operator regression to identify optimal predictive factors associated with APOs. Nine machine learning models were developed and subsequently underwent a comprehensive comparative evaluation of their predictive performance, leading to the identification of the optimal predictive model. SHAP values were generated to provide interpretable insights into model predictions.

**Results:**

A total of 288 patients were included in the study, 124 (43.06%) of whom had APOs. The extreme gradient boosting algorithm was shown to be the optimal model after a comparison of 9 different models utilizing various metrics. The SHAP analysis showed that crown-rump length at 6^+0^ to 8^+6^ weeks, the number of other drugs, the number of complications during pregnancy, gestational sac volume at 6^+0^ to 8^+6^ weeks, and yolk sac diameter change at 6^+0^ to 8^+6^ weeks were the key predictive factors affecting APOs.

**Conclusions:**

The study developed an interpretable predictive tool for APOs in patients with immune abnormalities, which may assist clinicians in making early intervention decisions.

## Introduction

When a woman becomes pregnant, the symptoms of autoimmune conditions may improve, worsen, or remain unchanged depending on her specific condition, and such conditions may complicate pregnancy as antibodies produced by the mother can enter the fetal system during development—for example, anti-Ro/SSA antibodies crossing the placenta and impacting the development of the fetal heart [1]. Prior studies have shown that systemic autoimmune conditions are associated with higher risks of adverse pregnancy outcomes (APOs), including spontaneous abortion, preeclampsia or eclampsia, intrauterine growth restriction, and stillbirth [2]. Moreover, reproductive failure in women has been associated with the presence of autoantibodies, including antiphospholipid antibodies and antinuclear antibodies, even in the absence of autoimmune diseases [3]. Hence, it is crucial to develop a prognostic diagnostic model for pregnant women with immune abnormalities or autoimmune diseases, which can predict the risk of subsequent APOs in the first trimester, so as to assist doctors in diagnosing and making informed decisions on timely immunosuppressive or anticoagulant therapy.

Clinical data have been collected and analyzed to identify risk factors for APOs and establish early predictive models. In a prospective study, Moyle et al [4] discovered that in pregnant individuals with antiphospholipid antibodies, with or without systemic lupus erythematosus, anti-β2 glycoprotein-I domain 1 and antiphosphatidylserine-prothrombin IgG are significant independent predictors of APOs and are strongly associated with lupus anticoagulant. Reis et al [5] constructed separate logistic regression models for outcomes, including gestational loss, preterm delivery, fetal growth restriction (FGR), and preeclampsia, and analyzed the predictors for each model. By synthesizing the results of these models, it was found that lupus flares during pregnancy were the most significant predictors of APOs. Prior studies predominantly developed predictive models based on baseline maternal information, previous manifestations, biochemical biomarkers, and medication use, while largely overlooking the predictive value of sonographic parameters for pregnancy outcomes. Gad et al [6] found that yolk sac size abnormalities serve as a reliable early predictor of pregnancy loss, detectable prior to fetal morphological assessment by ultrasound. Zhang et al [7] conducted a study to investigate whether the presence of endometrial cavity fluid (ECF) in infertile patients scheduled to undergo in vitro fertilization or intracytoplasmic sperm injection was associated with pregnancy outcomes. They found that the presence of ECF is an indicator of APOs, even when the fluid measures less than 3.5 mm in the anteroposterior dimension. Xu et al [8] investigated the association between crown-rump length (CRL) during the first trimester of pregnancy and neonatal outcomes. CRL was positively associated with birth length, birth weight, incidence of large-for-gestational-age infants, and preterm birth, while negatively associated with the incidence of small-for-gestational-age infants and neonatal intensive care unit admission. Although prenatal ultrasonography is integral to routine antenatal care, research into its value for predicting APOs in pregnancies with maternal autoimmune diseases and abnormal immune antibody profiles is markedly lacking. Notably, no systematic reviews or meta-analyses have synthesized the predictive value of sonographic parameters in this high-risk population, nor are there any validated, condition-specific predictive models integrating ultrasound markers with maternal immunological profiles to predict the risk of adverse outcomes. This paucity of consolidated evidence and dedicated clinical tools impedes the development of targeted antenatal surveillance protocols.

Current studies predominantly rely on conventional logistic regression models for predicting APOs. However, this methodology is inherently limited by its statistical assumptions, including linearity and observation independence requirements, which constrain the analysis to predefined predictor-outcome relationships while failing to capture complex nonlinear interactions. These limitations substantially compromise the models’ clinical predictive performance [9]. In contrast, machine learning (ML) has emerged as a powerful alternative, demonstrating superior performance in clinical outcome predictions. In contrast to conventional approaches, ML is capable of managing intricate interactions and nonlinear associations, which has contributed to its growing adoption in clinical research. In this field, the outcomes under investigation frequently rely on complex relationships among numerous factors [10]. Although ML models attain high levels of accuracy, the impact of individual variables on these models often remains unclear. This lack of transparency hinders the application of ML in clinical practice [11]. Shapley additive explanations (SHAP), a cutting-edge development aimed at enhancing the interpretability of tree-based models, utilizes a game theory–inspired approach. It consolidates the local contributions of individual features to account for the model’s overall performance on a global level. This method is regarded as more effective than other global approximation techniques. Moreover, the algorithm not only quantifies the importance of features throughout the model but also sheds light on how each feature functions in particular predictive outcomes [12].

**Figure 1.**
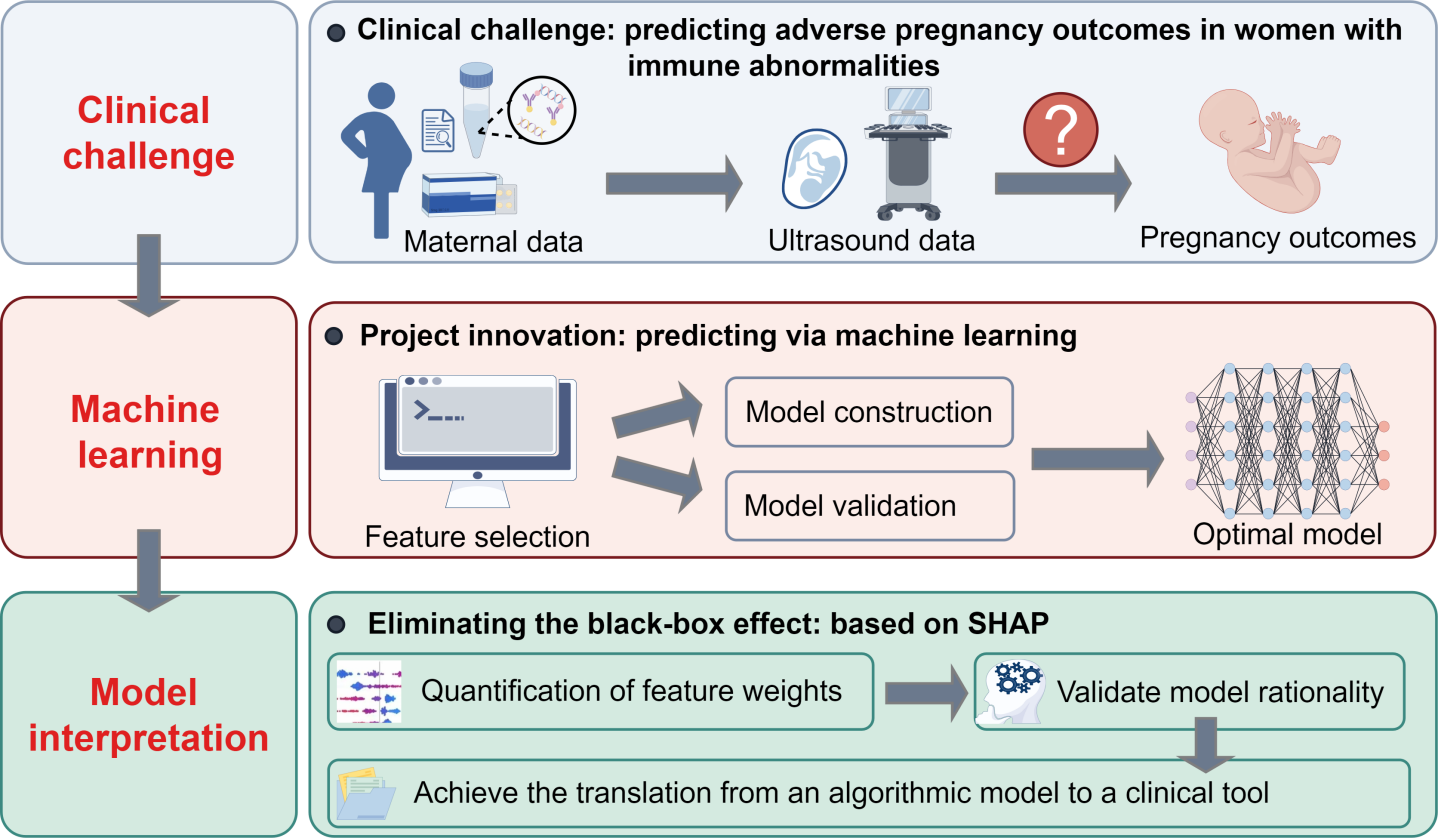
Graphical abstract of the study. SHAP: Shapley additive explanations.

Therefore, this study innovatively integrates first-trimester sonographic parameters with comprehensive clinical data to develop an ML-based predictive model for the early and precise risk assessment of APOs in patients with positive immune markers or diagnosed autoimmune diseases, enhances the clinical utility of the established model by employing SHAP analytical methods for model interpretability visualization, and further facilitates clinicians’ accurate identification of high-risk populations and timely clinical intervention through this interpretable ML framework. The graphical abstract is presented in Figure 1.

## Methods

**Figure 2.**
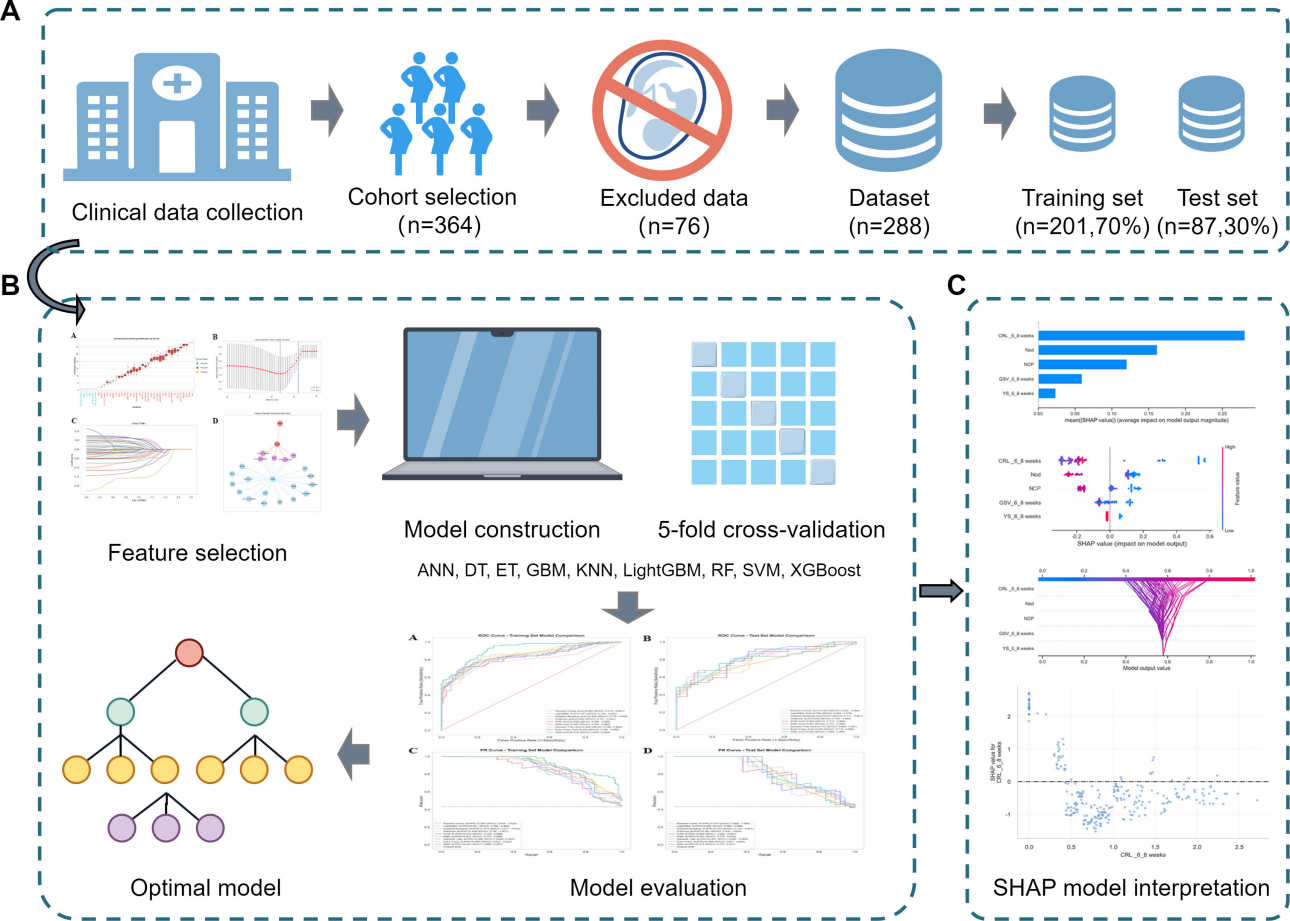
Flowchart of the predictive model. (A) Data collection, (B) model construction, and (C) model interpretation. ANN: artificial neural network; DT: decision tree; ET: extra tree; GBM: gradient boosting machine; KNN: k-nearest neighbor; LightGBM: light gradient boosting machine; RF: random forest; SHAP: Shapley additive explanations; SVM: support vector machine; XGBoost: extreme gradient boosting.

The overall workflow is illustrated in Figure 2. The completed TRIPOD+AI checklist, detailing the reporting of this prediction model study, is provided in Checklist 1.

### Ethical Considerations

This retrospective study was approved by the Ethics Committee of The First College of Clinical Medical Science, China Three Gorges University & Yichang Central People’s Hospital (approval number 2024-335-01) with a waiver of informed consent. The study was conducted in strict accordance with the requirements of the Declaration of Helsinki. All patient data were anonymized, access was limited to research team members only, and all data were handled in strict confidence to prevent any information disclosure. No compensation was provided to the participants.

### Data Acquisition

This retrospective study analyzed data from all first-trimester patients with positive immunological marker tests or a diagnosis of autoimmune diseases in the Obstetrics and Rheumatology departments of The First College of Clinical Medical Science of China Three Gorges University between January 2019 and October 2024. This study enrolled women aged 18 to 45 years with singleton pregnancies at 6^+0^ to 8^+6^ weeks of gestation (with gestational age correction when indicated), as confirmed by first-trimester ultrasonography, all of whom completed first-trimester immunological marker testing. To preserve data quality, patient records containing missing or anomalous data were excluded from the study. The stringent inclusion and exclusion criteria applied in this research were designed to guarantee the completeness and dependability of information pertaining to the included cases, thereby establishing a high-caliber database for assessing the risk of APOs in patients with autoimmune disorders. The inclusion criteria were as follows: (1) underwent first-trimester ultrasound and serological testing for autoimmune diseases, (2) age between 18 and 45 years, (3) history of autoimmune disease or abnormal autoimmune antibody levels, (4) singleton intrauterine pregnancy, (5) regular medication adherence as prescribed by physicians, and (6) availability of pregnancy outcome data. The exclusion criteria were as follows: (1) twin pregnancy patient, (2) noncompliance with the prescribed treatment plan, (3) lack of follow-up data on pregnancy outcomes, and (4) missing pregnancy details. Of the 364 patients retrieved from the electronic medical record system, 288 patients were enrolled in this study.

### Research Variables

To mitigate the impact of invalid variables on model calculations, the identification of scientifically legitimate variables is essential. By integrating ultrasound-specific domain knowledge and retrieving literature on predictive factors in PubMed, the following 39 research variables with potential discriminative ability were screened out: basic characteristics (age, number of previous miscarriages, and history of rheumatic immune disease), number of complications during pregnancy, abnormal antibody values, medication during pregnancy (which included the number of medications used, hydroxychloroquine, glucocorticoids, low-molecular-weight heparin, compound aspirin, progestogens, intravenous immunoglobulin, cyclosporine A, and the number of other drugs), and ultrasound measurement values at 6^+0^ to 8^+6^ weeks of gestation (yolk sac diameter change, gestational sac volume [GSV], CRL, and ECF).

This study employed APOs as the primary outcome variable, defined as the occurrence of any one of the following: miscarriage, preterm birth, biochemical pregnancy, low birth weight, fetal arrhythmia, neonatal asphyxia, or stillbirth [13].

### Feature Selection

To remove the impact of scale differences between variables and enhance the performance of the model and the comparability between features, the data were standardized using the *z* score standardization method, which is a commonly used and widely applied approach. During the data standardization process, the following coding criteria were implemented for yolk sac diameter: code 0, which indicates yolk sac diameter within a normal reference range (3‐6 mm); code 1, which signifies yolk sac enlargement (>6 mm); and code –1, which denotes yolk sac absence (nonvisualization on ultrasonography).

We employed a dual-feature selection approach combining the Least Absolute Shrinkage and Selection Operator (LASSO) regression and the Boruta algorithm to identify robust predictors associated with APOs. Using scikit-learn’s LassoCV in Python, we automatically selected the optimal regularization parameter alpha (λ_min) that minimizes the cross-validated mean squared error and then applied the 1 SE rule to determine a more conservative α value (λ_1se), yielding a sparser model and retaining features with nonzero coefficients from both models. Concurrently, we implemented the Boruta algorithm in Python using the Boruta-py package (maintained by Daniel Homola). The algorithm iteratively compares variable importance against randomly generated shadow features using a random forest (RF) classifier to identify statistically significant predictors [14]. This process continued until either feature importance rankings stabilized or a maximum of 100 iterations was reached. The final feature set comprised only those variables consistently selected by both LASSO and the Boruta algorithm, ensuring high-confidence predictors of APOs risk.

### Model Construction and Evaluation Metrics

We utilized 70% of the data for training the model through random sampling, reserving the remaining 30% for testing the model’s performance. Based on the features selected by Boruta and LASSO in the previous section, we developed 9 predictive models using artificial neural network (ANN), decision tree, extra tree, gradient boosting machine, k-nearest neighbor, light gradient boosting machine, RF, support vector machine, and extreme gradient boosting (XGBoost) algorithm. The Python scikit-learn library (version 1.3.0; originally created by David Cournapeau et al) was used to build the ML models. A combination of grid search with 5-fold cross-validation was employed to optimize the model’s hyperparameters. The predictive models were built on the training set, and the test set was validated on the best model. The bootstrap method was implemented with 1000 replications to derive the CI of the area under the receiver operating characteristic curve (AUC), accuracy, specificity, and *F*_1_-score for both the test and training sets. Given that our model’s primary objective is to accurately detect positive samples, often known as APOs, our main focus was on maximizing the recall value. Furthermore, we also generated the precision-recall curve and the area under the precision-recall curve (AUPRC) for each model.

### SHAP Model Interpretation

SHAP is a technique used to interpret predictions from complex ML models, especially those with numerous features [15]. It provides both cohort and patient-level insights by quantifying each feature’s contribution to predictions. To assess global feature importance, we aggregated and averaged the absolute SHAP values across all patients, where higher values indicate greater predictive influence. The SHAP summary plot visualizes feature importance and directional effects, where each point represents the SHAP value for an individual patient. The color gradient indicates relative feature values, with red corresponding to high values and blue to low values. The SHAP decision plot visualizes the relationship between individual patients’ feature-level contributions and the corresponding model predictions. By analyzing the trajectory patterns of the curves, we can observe feature-specific effects across different patients and quantitatively assess how each feature drives the final prediction outcomes. Dependence plots further elucidate how specific features influence the model output. The SHAP method was implemented in Python using SHAP version 0.47.2.

### Statistical Analysis

Statistical analyses were conducted using R (version 4.3.0) (R Core Team), with ML model development performed using scikit-learn in Python 3.12.7. Normality assessments were performed for continuous variables. Variables conforming to a normal distribution were represented as mean (SD) and analyzed using the *t* test. Nonnormally distributed data were presented as median (IQR; Q₁-Q₃) and were compared using the Mann-Whitney *U* test. Categorical data were expressed as frequencies and percentages (n, %) and analyzed using the *χ*² test or Fisher exact test. A *P* value of <.05 was considered to denote statistical significance.

## Results

### Clinical Characteristics of Patients

**Figure 3.**
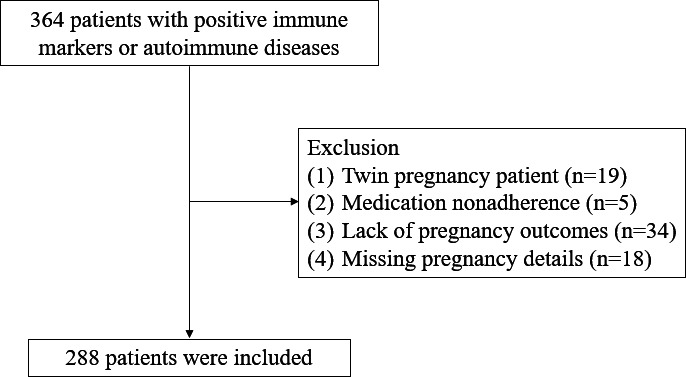
Specifics of the patient inclusion and exclusion processes.

A total of 288 patients were included in the study, divided into the No-APO group (n=164) and APO group (n=124). Among APO cases, the spectrum of adverse outcomes included 76 (26.39%) miscarriages, 22 (7.64%) preterm births, 17 (4.51%) biochemical pregnancies, 10 (3.45%) low birth weight, 3 (1.04%) fetal arrhythmia, 2 (0.07%) neonatal asphyxia, and 2 (0.07%) stillbirth. Some cases had 2 of the aforementioned adverse outcomes. The mean age of the patients was 31.04 (SD 5.37) years. A total of 177 (61.46%) patients were found to have autoimmune diseases. A comprehensive flowchart illustrating the inclusion and exclusion criteria is presented in Figure 3.

### Feature Selection

**Figure 4.**
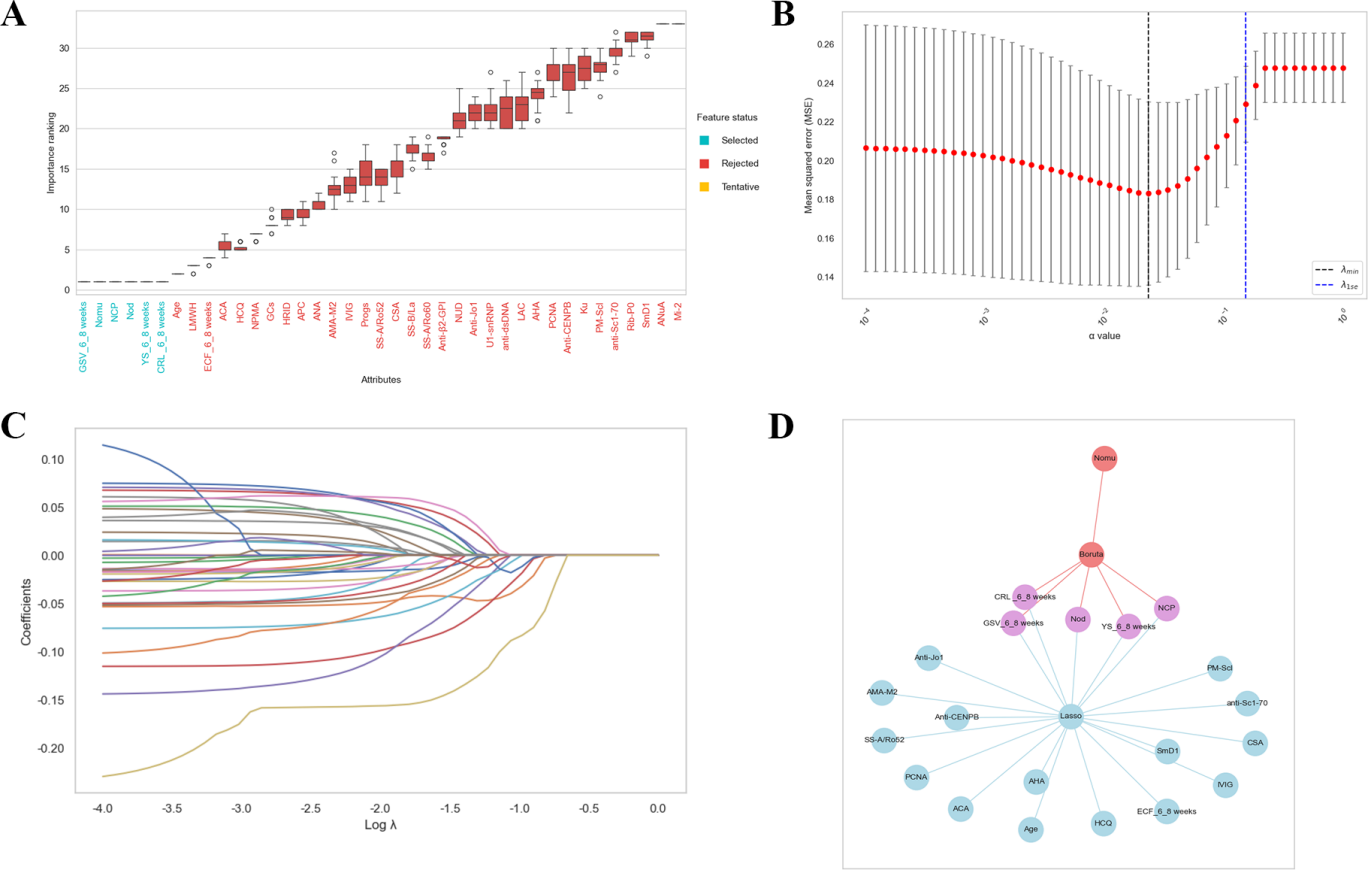
Feature selection. (A) Boruta, (B) feature screening based on the LASSO regression model, (C) the LASSO regression model coefficient path plot, and (D) common predictors between Boruta and LASSO. ACA: anti-cardiolipin antibody; AHA: anti-histone antibody; AMA-M2: anti-mitochondrial antibody M2; ANA: antinuclear antibodies; ANuA: anti-nucleosome antibody; Anti-β2-GPI: anti-β2-glycoprotein-I; Anti-CENPB: anti-centromere protein B antibody; Anti-dsDNA: anti-double-stranded DNA antibody; Anti-Jo1: anti-histidyl-tRNA synthetase; Anti-Scl-70: anti-topoisomerase I antibody; APC: compound aspirin; CRL-6-8 weeks: crown-rump length at 6^+0^ to 8^+6^ weeks; CSA: cyclosporine A; ECF-6‐8 weeks: endometrial cavity fluid at 6^+0^ to 8^+6^ weeks; GC: glucocorticoids; GSV-6‐8 weeks: gestational sac volume at 6^+0^ to 8^+6^ weeks; HCQ: hydroxychloroquine; HRID: history of rheumatic immune disease; IVIG: intravenous immunoglobulin; LAC: lupus anticoagulant testing (screening + confirmatory assays); LMWH: low-molecular-weight heparin; NCP: number of complications during pregnancy; Nod: number of other drugs; NPMA: number of previous miscarriages; PCNA: proliferating cell nuclear antigen antibody; PM-Scl: polymyositis-scleroderma antibody; Progs: progestogens; Rib-P0: ribosomal P0 protein antibody; SmD1: small nuclear ribonucleoprotein Sm D1; SS-A/Ro52: anti-Sjögren syndrome antigen A/Ro52; SS-A/Ro60: anti-Sjögren syndrome antigen A/Ro60 antibody; SS-B/La: Sjögren syndrome antigen B/La antibody; U1-snRNP: U1 small nuclear ribonucleoprotein antibody; YS-6-8 weeks: yolk sac diameter change at 6^+0^ to 8^+6^ weeks.

Participants were randomly allocated to either the training set (201/288, 70%) or the test set (87/288, 30%). Statistical analysis revealed no significant differences between the 2 groups (Table 1). Boruta is a feature selection algorithm rooted in the RF classifier. It works by repeatedly contrasting the importance of each variable with that of randomly permuted attributes to pinpoint variables with significant relevance. The selection results of the Boruta algorithm are summarized in Figure 4A. Variables having the box plot in green show that all predictors are important. If boxplots are in red, it shows they are rejected. If the box plots are in yellow, it indicates that they are tentative. The following features were associated with the risk of APOs: GSV at 6^+0^ to 8^+6^ weeks, number of medications used, number of complications during pregnancy, number of other drugs, yolk sac diameter change at 6^+0^ to 8^+6^ weeks, and CRL at 6^+0^ to 8^+6^ weeks. In addition, LASSO regression functions as a shrinkage estimation technique that achieves variable selection and complexity adjustment by constructing an optimization objective function that includes penalty terms. In this study, LASSO regression was utilized to identify characteristic factors such as GSV at 6^+0^ to 8^+6^ weeks, CRL at 6^+0^ to 8^+6^ weeks, ECF at 6^+0^ to 8^+6^ weeks, yolk sac diameter change at 6^+0^ to 8^+6^ weeks, anti-cardiolipin antibody, anti-topoisomerase I antibody, anti-Sjögren syndrome antigen A/Ro52 antibody, anti-mitochondrial antibody M2, anti-histidyl-tRNA synthetase antibody, proliferating cell nuclear antigen antibody, polymyositis-scleroderma antibody, anti-centromere protein B antibody, anti-histone antibody, small nuclear ribonucleoprotein Sm D1, age, number of complications during pregnancy, hydroxychloroquine, intravenous immunoglobulin, cyclosporine A, and number of other drugs (Figure 4B and C). Through a comparative analysis of LASSO regression and the Boruta algorithm for feature selection, we identified their commonly selected feature subset. These selected features were ultimately utilized in the construction of the model and consisted of GSV at 6^+0^ to 8^+6^ weeks, yolk sac diameter change at 6^+0^ to 8^+6^ weeks, CRL at 6^+0^ to 8^+6^ weeks, number of complications during pregnancy, and number of other drugs (Figure 4D). Using these 5 variables, we developed 9 ML models to predict the risk of APOs in individuals with positive immune markers or diagnosed autoimmune diseases.

**Table 1.** Baseline characteristics of study population.

Variable	Overall (N=288)	Test set^a^	Training set^b^	*P* value
Age, median (IQR)	31.00 (28.00-34.00)	31.00 (28.00-34.00)	31.00 (29.00-34.00)	.59
Number of previous miscarriages, median (IQR)	1.00 (0.00-2.00)	1.00 (0.00-2.00)	1.00 (0.00-2.00)	.40
Number of underlying diseases, median (IQR)	0.00 (0.00-0.00)	0.00 (0.00-0.00)	0.00 (0.00-0.00)	.007
Number of complications during pregnancy, median (IQR)	1.00 (0.00-2.00)	1.00 (0.00-2.00)	1.00 (0.00-2.00)	.19
Number of medications used, median (IQR)	5.00 (3.00-7.00)	5.00 (3.00-7.00)	5.00 (2.00-7.00)	.60
Number of other drugs, median (IQR)	2.00 (1.00-3.00)	2.00 (1.00-3.00)	2.00 (1.00-3.00)	.85
Gestational sac volume at 6^+0^ to 8^+6^ weeks, median (IQR)	4.87 (2.42-9.50)	4.60 (2.21-8.79)	5.10 (2.44-9.72)	.44
Crown-rump length at 6^+0^ to 8^+6^ weeks, median (IQR)	0.80 (0.45-1.24)	0.83 (0.56-1.18)	0.79 (0.43-1.26)	.54
Endometrial cavity fluid at 6^+0^ to 8^+6^ weeks, median (IQR)	0.00 (0.00-0.00)	0.00 (0.00-0.00)	0.00 (0.00-0.00)	.56
History of rheumatic immune disease, n (%)				.07
No	110 (38.19)	40 (45.98)	70 (34.83)	
Yes	178 (61.81)	47 (54.02)	131 (65.17)	
ACA^c^, n (%)				.02
Negative	226 (78.47)	76 (87.36)	150 (74.63)	
Positive	62 (21.53)	11 (12.64)	51 (25.37)	
Anti-β2-glycoprotein-I, n (%)				.23
Negative	249 (86.46)	72 (82.76)	177 (88.06)	
Positive	39 (13.54)	15 (17.24)	24 (11.94)	
ANA^d^, n (%)				.72
Negative	170 (59.03)	50 (57.47)	120 (59.70)	
Positive	118 (40.97)	37 (42.53)	81 (40.30)	
Lupus anticoagulant testing (screening+confirmatory assays), n (%)				>.99
Negative	275 (95.49)	83 (95.40)	192 (95.52)	
Positive	13 (4.51)	4 (4.60)	9 (4.48)	
Anti-Scl-70^e^, n (%)				.05
Negative	283 (98.26)	83 (95.40)	200 (99.50)	
Positive	5 (1.74)	4 (4.60)	1 (0.50)	
SS-A/Ro52^f^, n (%)				>.96
Negative	229 (79.51)	69 (79.31)	160 (79.60)	
Positive	59 (20.49)	18 (20.69)	41 (20.40)	
SS-A/Ro60^g^, n (%)				.55
Negative	198 (68.75)	62 (71.26)	136 (67.66)	
Positive	90 (31.25)	25 (28.74)	65 (32.34)	
U1-snRNP^h^, n (%)				.18
Negative	281 (97.57)	87 (100)	194 (96.52)	
Positive	7 (2.43)	0 (0)	7 (3.48)	
SS-B/La^i^, n (%)				.42
Negative	264 (91.67)	78 (89.66)	186 (92.54)	
Positive	24 (8.33)	9 (10.34)	15 (7.46)	
AMA-M2^j^, n (%)				.54
Negative	282 (97.92)	84 (96.55)	198 (98.51)	
Positive	6 (2.08)	3 (3.45)	3 (1.49)	
Anti-Jo1^k^, n (%)				.22
Negative	285 (98.96)	85 (97.70)	200 (99.50)	
Positive	3 (1.04)	2 (2.30)	1 (0.50)	
PCNA^l^, n (%)				>.99
Negative	285 (98.96)	86 (98.85)	199 (99)	
Positive	3 (1.04)	1 (1.15)	2 (1)	
PM-Scl^m^, n (%)				.56
Negative	285 (98.96)	87 (100)	198 (98.51)	
Positive	3 (1.04)	0 (0)	3 (1.49)	
Anti-CENPB^n^, n (%)				>.99
Negative	283 (98.26)	85 (97.70)	198 (98.51)	
Positive	5 (1.74)	2 (2.30)	3 (1.49)	
ANuA^o^, n (%)				.30
Negative	287 (99.65)	86 (98.85)	201 (100)	
Positive	1 (0.35)	1 (1.15)	0 (0)	
Anti-dsDNA^p^, n (%)				>.99
Negative	284 (98.61)	86 (98.85)	198 (98.51)	
Positive	4 (1.39)	1 (1.15)	3 (1.49)	
AHA^q^, n (%)				.51
Negative	286 (99.31)	86 (98.85)	200 (99.50)	
Positive	2 (0.69)	1 (1.15)	1 (0.50)	
Ku antibody, n (%)				>.99
Negative	287 (99.65)	87 (100)	200 (99.50)	
Positive	1 (0.35)	0 (0)	1 (0.50)	
Rib-P0^r^, n (%)				>.99
Negative	284 (98.61)	86 (98.85)	198 (98.51)	
Positive	4 (1.39)	1 (1.15)	3 (1.49)	
SmD1^s^, n (%)				>.99
Negative	287 (99.65)	87 (100)	200 (99.50)	
Positive	1 (0.35)	0 (0)	1 (0.50)	
Mi-2 antibody, n (%)				.30
Negative	287 (99.65)	86 (98.85)	201 (100)	
Positive	1 (0.35)	1 (1.15)	0 (0)	
HCQ^t^, n (%)				>.95
Not used	163 (56.60)	49 (56.32)	114 (56.72)	
Used	125 (43.40)	38 (43.68)	87 (43.28)	
GCs^u^, n (%)				.59
Not used	172 (59.72)	54 (62.07)	118 (58.71)	
Used	116 (40.28)	33 (37.93)	83 (41.29)	
LMWH^v^, n (%)				.29
Not used	126 (43.75)	34 (39.08)	92 (45.77)	
Used	162 (56.25)	53 (60.92)	109 (54.23)	
APC^w^, n (%)				.06
Not used	204 (70.83)	55 (63.22)	149 (74.13)	
Used	84 (29.17)	32 (36.78)	52 (25.87)	
Progs^x^, n (%)				.72
Not used	95 (32.99)	30 (34.48)	65 (32.34)	
Used	193 (67.01)	57 (65.52)	136 (67.66)	
IVIG^y^, n (%)				>.99
Not used	281 (97.57)	85 (97.70)	196 (97.51)	
Used	7 (2.43)	2 (2.30)	5 (2.49)	
CSA^z^, n (%)				.54
Not used	271 (94.10)	83 (95.40)	188 (93.53)	
Used	17 (5.90)	4 (4.60)	13 (6.47)	
Yolk sac diameter change at 6^+0^ to 8^+6^ weeks, n (%)				.92
Absent	40 (13.89)	13 (14.94)	27 (13.43)	
Enlarged diameter	9 (3.13)	3 (3.45)	6 (2.99)	
Normal diameter	239 (82.99)	71 (81.61)	168 (83.58)	

a Test set: n=87 (30%).

b Training set: n=201 (70%).

c ACA: anti-cardiolipin antibody.

d ANA: antinuclear antibodies.

e Anti-Scl-70: anti-topoisomerase I antibody.

f SS-A/Ro52: anti-Sjögren syndrome antigen A/Ro52.

g SS-A/Ro60: anti-Sjögren syndrome antigen A/Ro60 antibody.

h U1-snRNP: U1 small nuclear ribonucleoprotein antibody.

i SS-B/La: Sjögren syndrome antigen B/La antibody.

j AMA-M2: anti-mitochondrial antibody M2.

k Anti-Jo1: anti-histidyl-tRNA synthetase.

l PCNA: proliferating cell nuclear antigen antibody.

m PM-Scl: polymyositis-scleroderma antibody.

n Anti-CENPB: anti-centromere protein B antibody.

o ANuA: anti-nucleosome antibody.

p Anti-dsDNA: anti-double-stranded DNA antibody.

q AHA: anti-histone antibody.

r Rib-P0: ribosomal P0 protein antibody.

s SmD1: small nuclear ribonucleoprotein Sm D1.

t HCQ: hydroxychloroquine.

u GCs: glucocorticoids.

v LMWH: low-molecular-weight heparin.

w APC: compound aspirin.

x Progs: progestogens.

y IVIG: intravenous immunoglobulin.

z CSA: cyclosporine A.

### Model Construction and Evaluation

**Figure 5.**
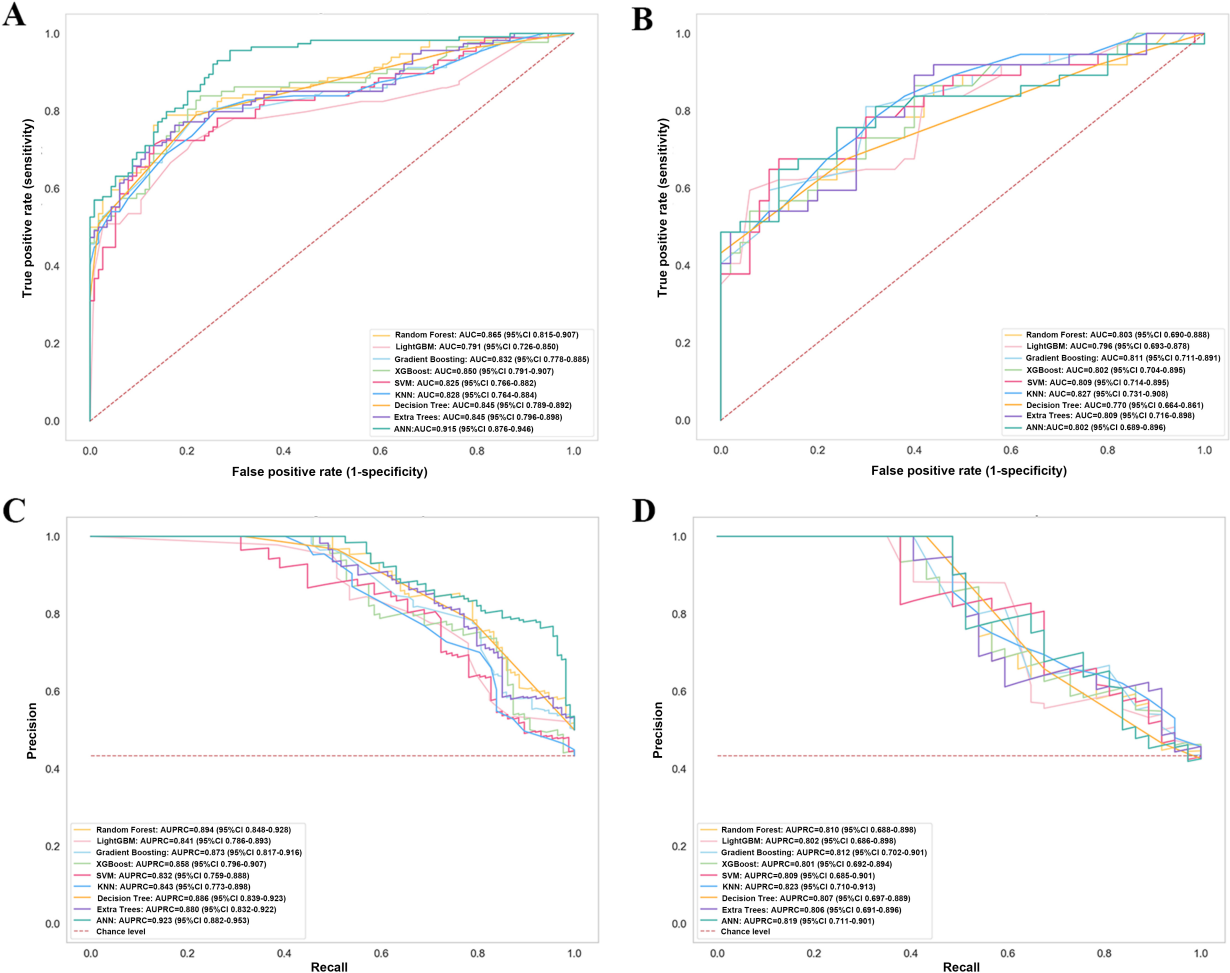
(A, B) Receiver operating characteristic (ROC) curves for the machine learning (ML) models in the training and test datasets and (C, D) precision-recall (PR) results for the ML models in the training and test datasets. ANN: artificial neural network; AUPRC: area under the precision-recall curve; DT: decision tree; ET: extra tree; GBM: gradient boosting machine; KNN: k-nearest neighbor; LightGBM: light gradient boosting machine; RF: random forest; SVM: support vector machine; XGBoost: extreme gradient boosting.

In this study, 9 different ML techniques were used to establish the model: ANN, decision tree, extra tree, gradient boosting machine, k-nearest neighbor, light gradient boosting machine, RF, support vector machine, and XGBoost. The performance of the 9 models in terms of AUC and AUPRC for both the training cohort and the test cohort is displayed in Figure 5. The accuracy, specificity, recall, and *F*_1_-score are compared in Table 2. Out of the 9 models, the ANN, RF, and XGBoost models demonstrated superior AUC performance (ANN: 0.915, 95% CI 0.876‐0.946; RF: 0.865, 95% CI 0.815‐0.907; XGBoost: 0.850, 95% CI 0.791‐0.907). The AUPRC was used to further compare the performances of the ML models because the AUPRC is an evaluation metric focused on positive class performance, which quantifies the overall trade-off between precision and recall, providing a critical performance indicator for cost-sensitive tasks. In both the training and test sets, the XGBoost model demonstrated balanced performance in AUPRC, with values of 0.858 (95% CI 0.796‐0.907) in the training set and 0.801 (95% CI 0.692‐0.894) in the test set. Additionally, the model maintained high recall across both datasets, indicating its stable discriminative efficacy and clinical utility in the detection of the target disease. Therefore, a downstream analysis was performed using the XGBoost model.

**Table 2.** Performance metrics for prediction models in the training set and the test set.

Model	Accuracy (95% CI)	Specificity (95% CI)	Recall (95% CI)	*F*_1_-score (95% CI)
Training set				
ANN^a^	0.790 (0.702-0.816)	0.798 (0.720-0.871)	0.769 (0.636-0.798)	0.749 (0.682-0.809)
DT^b^	0.798 (0.746-0.851)	0.781 (0.704-0.855)	0.816 (0.745-0.882)	0.802 (0.739-0.854)
ET^c^	0.785 (0.727-0.844)	0.763 (0.682-0.840)	0.813 (0.737-0.892)	0.771 (0.700-0.831)
GBM^d^	0.820 (0.761-0.873)	0.851 (0.780-0.912)	0.780 (0.685-0.862)	0.793 (0.721-0.852)
KNN^e^	0.724 (0.667-0.781)	0.752 (0.676-0.806)	0.765 (0.671-0.869)	0.727 (0.654-0.781)
LightGBM^f^	0.753 (0.687-0.808)	0.692 (0.603-0.781)	0.813 (0.727-0.889)	0.767 (0.694-0.826)
RF^g^	0.789 (0.737-0.838)	0.763 (0.685-0.839)	0.816 (0.742-0.886)	0.745 (0.635-0.850)
SVM^h^	0.765 (0.724-0.833)	0.705 (0.696-0.931)	0.825 (0.600-0.879)	0.743 (0.687-0.818)
XGBoost^i^	0.843 (0.726-0.856)	0.858 (0.671-0.905)	0.828 (0.698-0.924)	0.796 (0.722-0.868)
Test set				
ANN	0.716 (0.676-0.897)	0.720 (0.656-0.944)	0.697 (0.611-0.889)	0.678 (0.647-0.870)
DT	0.723 (0.641-0.805)	0.740 (0.617-0.857)	0.676 (0.605-0.828)	0.667 (0.620-0.781)
ET	0.701 (0.598-0.793)	0.720 (0.593-0.840)	0.676 (0.518-0.821)	0.658 (0.523-0.767)
GBM	0.744 (0.609-0.793)	0.820 (0.592-0.836)	0.695 (0.618-0.829)	0.747 (0.617-0.792)
KNN	0.682 (0.621-0.793)	0.692 (0.600-0.833)	0.686 (0.618-0.814)	0.685 (0.626-0.775)
LightGBM	0.678 (0.598-0.793)	0.640 (0.589-0.837)	0.749 (0.605-0.833)	0.732 (0.665-0.769)
RF	0.720 (0.609-0.805)	0.720 (0.596-0.840)	0.769 (0.617-0.883)	0.705 (0.644-0.771)
SVM	0.713 (0.653-0.874)	0.660 (0.787-0.960)	0.784 (0.612-0.815)	0.699 (0.600-0.838)
XGBoost	0.790 (0.658-0.905)	0.804 (0.798-0.893)	0.811 (0.676-0.933)	0.758 (0.664-0.795)

a ANN: artificial neural network.

b DT: decision tree.

c ET: extra tree.

d GBM: gradient boosting machine.

e KNN: k-nearest neighbor.

f LightGBM: light gradient boosting machine.

g RF: random forest.

h SVM: support vector machine.

i XGBoost: extreme gradient boosting.

### SHAP Model Interpretation

**Figure 6.**
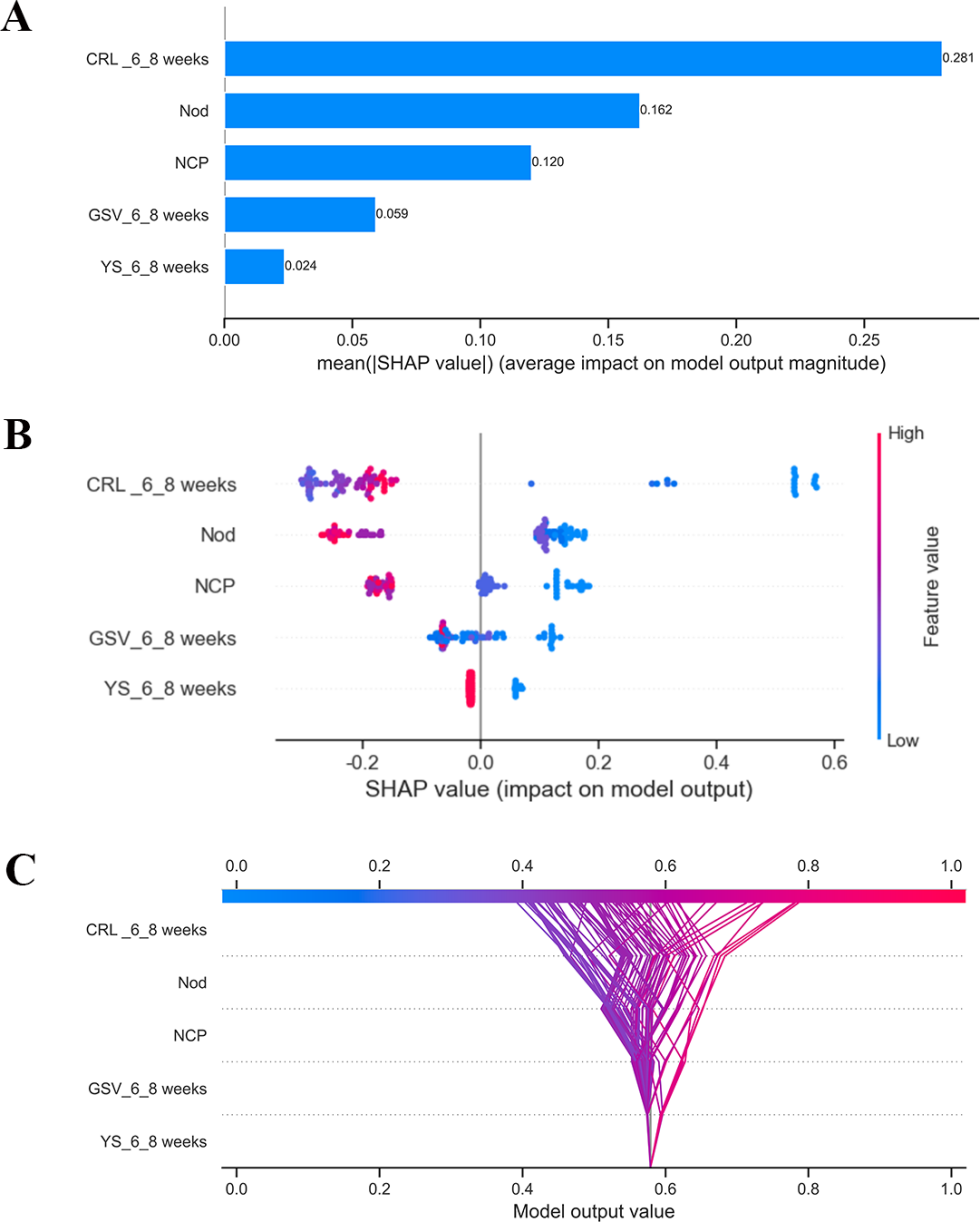
Variable contribution to the model by Shapley additive explanations (SHAP) values. (A) SHAP bar chart and (B) SHAP summary plot Each dot represents a SHAP value for a feature per patient. The x-axis is the SHAP value; color, from red to blue, indicates feature values from high to low. (C) SHAP decision plot. CRL-6‐8 weeks: crown-rump length at 6^+0^ to 8^+6^ weeks; GSV-6‐8 weeks: gestational sac volume at 6^+0^ to 8^+6^ weeks; NCP: number of complications during pregnancy; Nod: number of other drugs; YS-6‐8 weeks: yolk sac diameter change at 6^+0^ to 8^+6^ weeks.

The SHAP feature importance for the XGBoost model is shown in Figure 6A. The features assessed are arranged in descending order according to their mean absolute SHAP values, which reflect the degree of their impact on the model’s predictions. The specific ranking is as follows: CRL at 6^+0^ to 8^+6^ weeks, number of other drugs, number of complications during pregnancy, GSV at 6^+0^ to 8^+6^ weeks, and yolk sac diameter change. The SHAP summary plot of the XGBoost model shows the impact of features on the prediction model (Figure 6B). Based on the prediction model, a higher SHAP value of a feature indicates a greater likelihood of APOs. For example, patients with smaller GSV at 6^+0^ to 8^+6^ weeks are more likely to experience APOs compared to those with larger volumes during pregnancy. Moreover, the SHAP decision plot visually delineates the contribution of each clinical feature to individualized predictive outcomes. Through ANOVA trends in feature contribution values, the distinct differential impact patterns of specific clinical variables across different patients can be reliably identified (Figure 6C).

**Figure 7.**
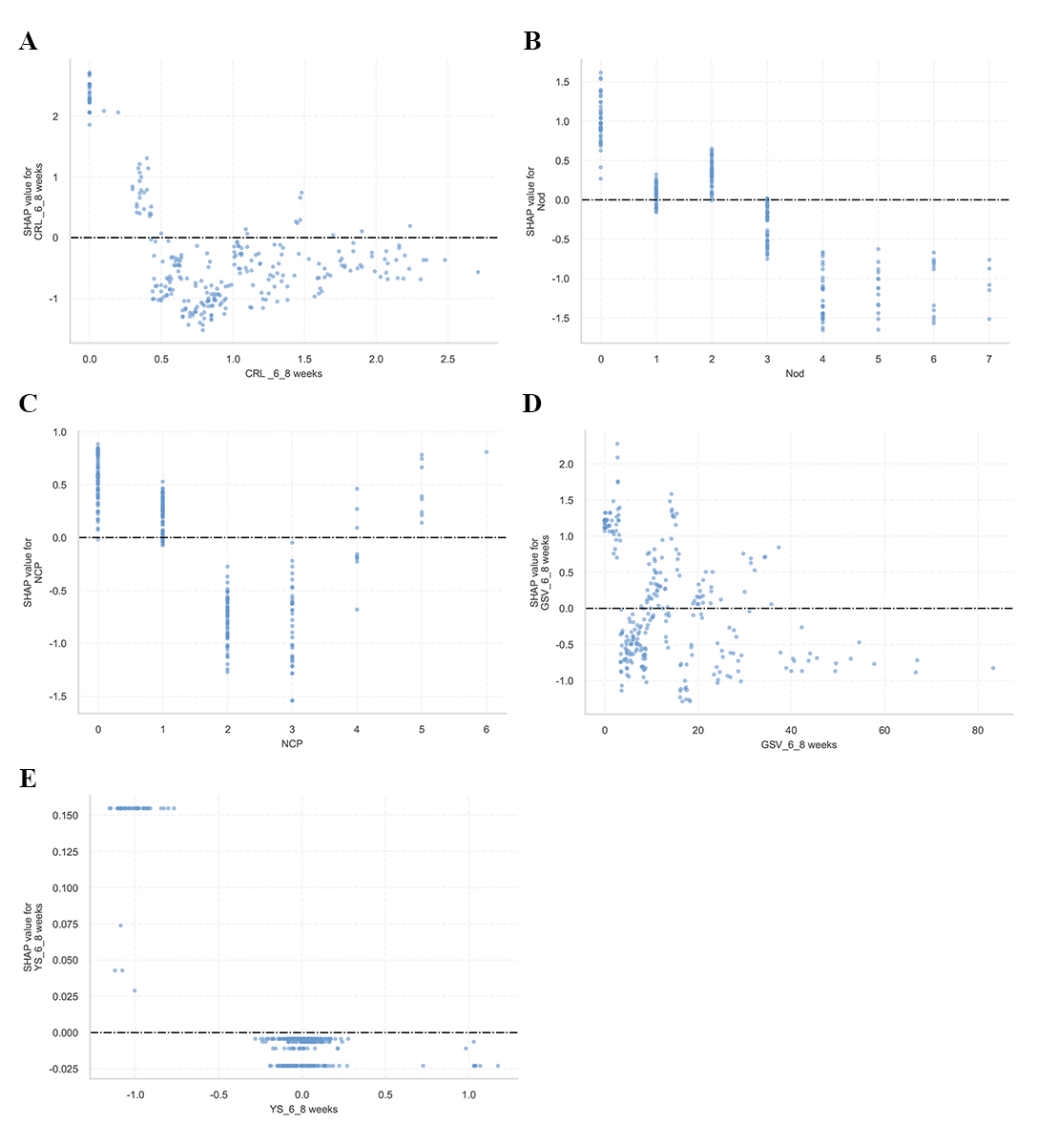
Shapley additive explanations (SHAP) dependence plots of model features: (A) CRL-6‐8weeks: crown-rump length at 6^+0^ to 8^+6^ weeks; (B) Nod: number of other drugs; (C) NCP: number of complications during pregnancy; (D) GSV-6‐8 weeks: gestational sac volume at 6^+0^ to 8^+6^ weeks; (E) YS-6‐8 weeks: yolk sac diameter change at 6^+0^ to 8^+6^ weeks. The y-axis shows features’ SHAP values; the x-axis displays specific feature values. Each dot represents a patient’s feature-specific SHAP value, with values above 0 indicating heightened adverse pregnancy outcome (APO) risk.

Additionally, the SHAP dependence plot illustrates how a feature influences the predictive outcomes of the XGBoost model (Figure 7). Patients with decreased CRL at 6^+0^ to 8^+6^ weeks and the number of other drugs (decrease in the x-axis value) were associated with a higher SHAP value, indicating a higher likelihood of APOs (increase in the y-axis value). Additionally, a larger GSV at 6^+0^ to 8^+6^ weeks was associated with lower SHAP values, suggesting a reduced likelihood of APOs. Conversely, the absence of a yolk sac during the same gestational period correlated with higher SHAP values, indicating a significantly elevated risk.

### Explanation of the ML Model at the Patient Level

**Figure 8.**
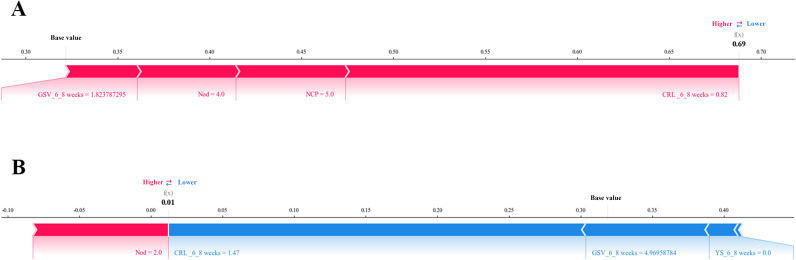
Shapley additive explanations (SHAP) force plot of (A) patient A (true positive) and (B) patient B (true negative). The color represents the contributions of each feature, with red being positive and blue being negative. The length of the color bar represents the contribution strength. CRL-6‐8weeks: crown-rump length at 6^+0^ to 8^+6^ weeks; GSV-6‐8 weeks: gestational sac volume at 6^+0^ to 8^+6^ weeks; NCP: number of complications during pregnancy; Nod: number of other drugs; YS-6‐8 weeks: yolk sac diameter change at 6^+0^ to 8^+6^ weeks (code 0 indicates yolk sac diameter within the normal reference range; code 1 signifies yolk sac enlargement; code −1 denotes yolk sac absence).

To evaluate the contributions of features for individual patients using the XGBoost model, we applied the SHAP method to explain the individual predictions for 2 patients. The color represents the contributions of each feature, with red being positive and blue being negative. The length of the color bar represents the contribution strength. For patient A, who was in the “true positive” group, the XGBoost model predicted a 69% probability of APOs (Figure 8A). For patient B, who was in the “true negative“ group, the XGBoost model predicted a 1% probability of APOs (Figure 8B).

Patient A, a 27-year-old female with a positive antinuclear antibody, was clinically diagnosed with antiphospholipid syndrome complicated by thrombotic predisposition. Transvaginal ultrasonography during early pregnancy (6^+0^ to 8^+6^ weeks of gestation) demonstrated the following parameters: CRL 0.82 cm, GSV 1.82 cm³, with normal yolk sac morphology. The pregnancy was complicated by multiple comorbidities, including gestational diabetes mellitus, hypothyroidism, anemia, and hypoproteinemia. Despite active symptomatic supportive therapy and standardized pregnancy-preserving interventions, the pregnancy ultimately resulted in preterm delivery.

Patient B, a 28-year-old female with a positive SS-B/La antibody (without confirmed autoimmune disease), during early pregnancy (6^+0^ to 8^+6^ weeks), underwent a transvaginal ultrasound that showed parameters consistent with gestational age: CRL 1.47 cm, GSV 4.97 cm³, and normal yolk sac morphology. The patient reported the use of 2 additional medication categories during pregnancy.

## Discussion

### Principal Findings

This study focuses on using ML models to predict APOs in patients with abnormal immune indices or autoimmune diseases during early pregnancy. We evaluated 9 ML methods for prediction, with the XGBoost model demonstrating the highest performance. Additionally, we utilized the SHAP approach to explore interpretations of the XGBoost model. The most important features were the CRL at 6^+0^ to 8^+6^ weeks, number of other drugs, number of complications during pregnancy, GSV at 6^+0^ to 8^+6^ weeks, and yolk sac diameter change at 6^+0^ to 8^+6^ weeks.

In previous clinical studies, variables predominantly focused on clinical demographic characteristics, clinical history, baseline clinical and laboratory indices, as well as medication and disease activity markers but overlooked the impact of first-trimester ultrasound parameters on APOs. Fazzari et al [16] developed multiple predictive models incorporating 41 clinical parameters (including lupus serological markers, antiphospholipid antibody assays, disease activity indices, etc) obtained during early gestation to assess APOs in systemic lupus erythematosus patients. The comparative analysis demonstrated that the Super Learner ensemble learning model achieved optimal predictive performance, with an AUC of 0.77. Jiang et al [17] utilized mean arterial pressure, prepregnancy hypertension, positive aCL-IgM, 24-hour urinary protein, serum albumin, history of hematological diseases, and serum uric acid, achieving an AUC of 0.975 (95% CI 0.958‐0.992) in a single-center sample of 587 cases. However, substantial evidence indicates that first-trimester ultrasound parameters, such as yolk sac diameter, GSV, and CRL, are significantly associated with APOs. Building upon this foundation, Pavanya et al [18,19] have demonstrated the value of both traditional and novel assessments. Their work not only identified key birthweight-related factors, including CRL and nuchal translucency thickness, but also developed an explainable artificial intelligence model that predicts early FGR by quantifying fetal movements from routine first-trimester ultrasound scans. The accurate measurement of foundational biometrics, such as CRL and nuchal translucency, serves as the critical baseline for such integrated risk assessment. Roelants et al [20] conducted a study with a sample size of 918 patients, utilizing virtual reality software on 3D ultrasound scans to measure CRL and embryonic volume (EV) at 7, 9, and 11 weeks of gestation. The study found that larger embryonic CRL and EV are associated with lower odds of adverse birth outcomes, especially small for gestational age (SGA).

We constructed a predictive model utilizing ML techniques that integrate sonographic parameters. Among the models developed, the XGBoost model yielded an AUC of 0.850 (95% CI 0.791‐0.907) and an AUPRC of 0.858 (95% CI 0.796‐0.907). In the test dataset, the XGBoost model also surpassed other ML models, achieving an AUC of 0.802 (95% CI 0.704‐0.895) and an AUPRC of 0.801 (95% CI 0.692‐0.894). For the purpose of interpreting our XGBoost model, we employed SHAP summary plots and dependence plots to identify the predictive factors of APOs, whereas SHAP force plots were used to analyze individual patient cases. The SHAP results showed that CRL at 6^+0^ to 8^+6^ weeks, number of other drugs, number of complications during pregnancy, GSV at 6^+0^ to 8^+6^ weeks, and yolk sac diameter change, which can be easily evaluated in clinical practice, are important features for the ML model to predict APOs.

Several sonographic parameters have been reported as possible predictors of APOs. For instance, EV has been associated with miscarriage and SGA outcomes and is also linked to maternal risk factors, such as smoking and vitamin B12 levels [21]. More specifically, an SGA GSV is a significant sonographic predictor of miscarriage, likely resulting from impaired placentation [22]. The first-trimester CRL is a well-established predictor: a small CRL (<10th percentile) significantly increases the risk of delivering an SGA neonate and preterm birth before 34 weeks, whereas a large CRL (≥90th percentile) is strongly associated with large-for-gestational-age outcomes [23]. Indeed, its association with birth weight is so strong that a small first-trimester CRL is a key indicator for subsequent low birth weight [24]. Notably, the SHAP dependence plot shows the effect of sonographic parameters on the XGBoost prediction. The yolk sac acts as the primary route of exchange between the human embryo and the mother before placental circulation is established. The yolk sac is a critical landmark that identifies a true gestational sac. In fact, it is the first conceptional structure that is usually visualized by transvaginal sonography when a gestational sac measures above 8 mm [25]. There have been conflicting results regarding the yolk sac. Some have suggested that the yolk sac diameter is not significantly associated with miscarriage [26], but others have demonstrated that the absence of a yolk sac or a small yolk sac is a significant predictor of miscarriage [27]. Our results demonstrate that the absence of a yolk sac is associated with an increased risk of APOs, a finding that corroborates previous research observations. Previous studies have highlighted that comorbidities during pregnancy, such as hypertensive disorders of pregnancy in the setting of chronic hypertension, are associated with the highest odds of serious consequences on the pregnant person and neonate [28]. Consequently, there exists a positive correlation between the number of antenatal comorbidities and the risk of APOs. However, our study found that APOs were associated with either ≤1 or >5 pregnancy complications, whereas the increased use of other medications was inversely correlated with APOs risk. Although clinical practice generally suggests that a higher burden of comorbidities portends a poorer prognosis, our data demonstrated that APO risk was significantly reduced in cases with 2 to 3 obstetric complications, as well as with the increased use of pregnancy complication–related medications and tocolytic agents. This observation may be explained by enhanced clinical management in high-risk pregnancies, including more intensive prenatal monitoring, optimized therapeutic interventions, and stricter medication supervision. Furthermore, potential confounding factors, including disease-disease interactions and the study’s limited sample size, may have contributed to model overfitting, potentially affecting outcome prediction accuracy.

### Limitation of the Study

This study has some limitations. First, the single-center design of this study may introduce selection bias, as the sample representativeness could be compromised by institution-specific clinical protocols that may influence data generation. Multicenter validation with diverse patient populations is required to enhance model generalizability. Second, APOs are a composite outcome, and each subtype (such as miscarriage, fetal growth restriction, and preterm birth) may have unique predictors. Future studies could develop subtype-specific prediction models and compare differences in feature importance. Third, the homogeneity of the validation data (originating from 1 institution) poses a threat to external validity. Prospective validation in independent, diverse cohorts is needed to assess performance across broader clinical spectra and populations.

### Conclusions

ML models utilizing easily available clinical data and sonographic parameters can predict APOs in individuals with positive immune markers or diagnosed autoimmune diseases during early pregnancy. The SHAP method offers clinically interpretable explanations for ML predictions, identifying patient-specific risk factors underlying APOs through intuitive visualizations. These insights directly support personalized obstetric counseling and facilitate targeted clinical interventions to mitigate pregnancy risks.

## Supplementary material

10.2196/84087 Checklist 1 TRIPOD+AI checklist for machine learning prediction model.

## Abbreviations

ANN: artificial neural network
APO: adverse pregnancy outcome
AUC: area under the receiver operating characteristic curve
AUPRC: area under the precision-recall curve
CRL: crown-rump length
ECF: endometrial cavity fluid
EV: embryonic volume
FGR: fetal growth restriction
GSV: gestational sac volume
LASSO: Least Absolute Shrinkage and Selection Operator
ML: machine learning
RF: random forest
SGA: small-for-gestational age
SHAP: Shapley additive explanations
XGBoost: extreme gradient boosting

## References

[1] Singh M, Wambua S, Lee SI (2024). Autoimmune diseases and adverse pregnancy outcomes: an umbrella review. BMC Med.

[2] Keum H, Bermas B, Patel S, Jacobe HT, Chong BF (2024). Patients with autoimmune skin diseases are at increased risk of adverse pregnancy outcomes. Am J Obstet Gynecol MFM.

[3] Cavalcante MB, Sarno M, Barini R (2025). Immune biomarkers in cases of recurrent pregnancy loss and recurrent implantation failure. Minerva Obstet Gynecol.

[4] Moyle KA, Branch DW, Peterson LK (2025). Association between novel antiphospholipid antibodies and adverse pregnancy outcomes. Obstet Gynecol.

[5] Palma Dos Reis CR, Cardoso G, Carvalho C, Nogueira I, Borges A, Serrano F (2020). Prediction of adverse pregnancy outcomes in women with systemic lupus erythematosus. Clin Rev Allergy Immunol.

[6] Gad HF, Salman MM, Koleib MH, Nada AI, Daoud MA (2024). Correlation between diameter of yolk sac on transvaginal ultrasonography at 6-12 weeks of gestation and adverse pregnancy outcomes. JBRA Assist Reprod.

[7] Zhang WX, Cao LB, Zhao Y (2021). Endometrial cavity fluid is associated with deleterious pregnancy outcomes in patients undergoing *in vitro* fertilization/intracytoplasmic sperm injection: a retrospective cohort study. Ann Transl Med.

[8] Xu Y, Ni M, Zhang Q, Zhao J, Tang Z, Liu Z (2022). Correlation between crown-rump length in the first trimester of pregnancy and neonatal outcomes. BMC Pediatr.

[9] Song X, Liu X, Liu F, Wang C (2021). Comparison of machine learning and logistic regression models in predicting acute kidney injury: a systematic review and meta-analysis. Int J Med Inform.

[10] Hsu WH, Ko AT, Weng CS (2023). Explainable machine learning model for predicting skeletal muscle loss during surgery and adjuvant chemotherapy in ovarian cancer. J Cachexia Sarcopenia Muscle.

[11] Guan C, Gong A, Zhao Y (2024). Interpretable machine learning model for new-onset atrial fibrillation prediction in critically ill patients: a multi-center study. Crit Care.

[12] Qi X, Wang S, Fang C, Jia J, Lin L, Yuan T (2025). Machine learning and SHAP value interpretation for predicting comorbidity of cardiovascular disease and cancer with dietary antioxidants. Redox Biol.

[13] Huang Y, Xu J, Peng B, Zhang W (2023). Risk factors for adverse pregnancy outcomes in Chinese women: a meta-analysis. PeerJ.

[14] Wei Z, Li M, Zhang C, Miao J, Wang W, Fan H (2024). Machine learning-based predictive model for post-stroke dementia. BMC Med Inform Decis Mak.

[15] Jiang C, Xiu Y, Qiao K, Yu X, Zhang S, Huang Y (2022). Prediction of lymph node metastasis in patients with breast invasive micropapillary carcinoma based on machine learning and SHapley Additive exPlanations framework. Front Oncol.

[16] Fazzari MJ, Guerra MM, Salmon J, Kim MY (2022). Adverse pregnancy outcomes in women with systemic lupus erythematosus: can we improve predictions with machine learning?. Lupus Sci Med.

[17] Jiang M, Wang Y, Fu Q, Lin S, Wu J, Di W (2020). Preeclampsia risk prediction model for Chinese pregnant patients with systemic lupus erythematosus. Arthritis Care Res (Hoboken).

[18] Pavanya M, Chadaga K, J V (2025). Prediction of birthweight with early and mid-pregnancy antenatal markers utilising machine learning and explainable artificial intelligence. Sci Rep.

[19] Pavanya M, Chadaga K, J V, Vasudeva A, Rao BK, Bhat SK (2025). A model of the first trimester evaluation of foetal movements and their outcomes via explainable artificial intelligence: a multicentric study. Healthc Technol Lett.

[20] Roelants JA, Vermeulen MJ, Willemsen SP (2024). Embryonic size and growth and adverse birth outcomes: the Rotterdam Periconception Cohort. Hum Reprod.

[21] Bastiaansen WAP, Klein S, Hojeij B (2025). Automatic human embryo volume measurement in first trimester ultrasound from the Rotterdam Periconception Cohort: quantitative and qualitative evaluation of artificial intelligence. J Med Internet Res.

[22] Wie JH, Choe S, Kim SJ, Shin JC, Kwon JY, Park IY (2015). Sonographic parameters for prediction of miscarriage: role of 3-dimensional volume measurement. J Ultrasound Med.

[23] Kwak DW, Yang JI, Song KH (2022). Prediction of adverse pregnancy outcomes using crown-rump length at 11 to 13 + 6 weeks of gestation. J Ultrasound Med.

[24] Patel S, Sarkar A, Pushpalatha K (2022). A prospective study on correlation of first trimester crown-rump length with birth weight. Cureus.

[25] Suguna B, Sukanya K (2019). Yolk sac size & shape as predictors of first trimester pregnancy outcome: a prospective observational study. J Gynecol Obstet Hum Reprod.

[26] Tan S, Gülden Tangal N, Kanat-Pektas M (2014). Abnormal sonographic appearances of the yolk sac: which can be associated with adverse perinatal outcome?. Med Ultrason.

[27] Marin M, Pătru CL, Manolea MM (2021). Can ultrasound analysis of the yolk sac be a predictor of pregnancy outcome?. Curr Health Sci J.

[28] Sweeney LC, Lundsberg LS, Culhane JF, Partridge C, Son M (2024). Co-existing chronic hypertension and hypertensive disorders of pregnancy and associated adverse pregnancy outcomes. J Matern Fetal Neonatal Med.

